# *Mulinum crassifolium* Phil; Two New Mulinanes, Gastroprotective Activity and Metabolomic Analysis by UHPLC-Orbitrap Mass Spectrometry

**DOI:** 10.3390/molecules24091673

**Published:** 2019-04-28

**Authors:** Carlos Areche, Ronald Fernandez-Burgos, Teresa Cano de Terrones, Mario Simirgiotis, Olimpo García-Beltrán, Jorge Borquez, Beatriz Sepulveda

**Affiliations:** 1 Departamento de Química, Facultad de Ciencias, Universidad de Chile, Casilla 653, Santiago, Chile; areche@uchile.cl (C.A.); rfmaximiliano@gmail.com (R.F.-B.); 2 Departamento de Química, Facultad de Ciencias Naturales y Formales, Universidad Nacional de San Agustín, Arequipa 68513, Peru; dcanof@unsa.edu.pe; 3 Instituto de Farmacia, Facultad de Ciencias, Universidad Austral de Chile, Valdivia 5090000, Chile; mario.simirgiotis@gmail.com; 4 Facultad de Ciencias Naturales y Matemáticas, Universidad de Ibagué, Carrera 22 calle 67, Ibagué 730002, Colombia; jose.garcia@unibague.edu.co; 5 Departamento de Química, Facultad de Ciencias Básicas, Universidad de Antofagasta, Av Coloso S-N, Antofagasta 1240000, Chile; jorge.borquez@uantof.cl; 6 Departamento de Ciencias Químicas, Universidad Andrés Bello, Campus Viña del Mar, Quillota 980, Viña del Mar, Chile

**Keywords:** diterpenoids, Mulinanes, *Mulinum crassifolium*, UHPLC-MS, Orbitrap, secondary metabolites

## Abstract

*Mulinum crassifolium* Phil. (Apiaceae) is an endemic shrub from Chile commonly used as infusion in traditional medicine to treat diabetes, bronchial and intestinal disorders and stomach ailments, including ulcers. From the EtOAc extract of this plant, the new mulinane-type diterpenoids **3** and **5** were isolated along with three known diterpenoids. The gastroprotective effect of the infusion of the plant was assayed to support the traditional use and a fast HPLC analysis using high resolution techniques was performed to identify the bioactive constituents. The EtOAc extract and the edible infusion showed gastroprotective effect at 100 mg/kg in the HCl/EtOH induced gastric ulcer model in mice, reducing lesions by 33% and 74%, respectively. Finally, a metabolomic profiling based on UHPLC-ESI-MS/HRMS of the edible infusion was performed and thirty-five compounds were tentatively identified including quercetin, caffeic acid, apigenine glucoside, *p*-coumaric acid, chlorogenic acids, and caffeoylquinic acids, which have been associated previously with gastroprotective and antiulcer properties. This scientific evidence can support the contribution of polyphenols in the gastroprotective activity of the edible infusion of this plant, and can validate at least in part, its ethnopharmacological use.

## 1. Introduction

*Mulinum crassifolium* (Apiaceae) is an endemic shrub confined to the north of Chile and commonly known as “chuquican” or “sucurco”. The whole plant is used as infusions in traditional medicine to treat diabetes, bronchial and intestinal disorders, and stomach sickness [1]. Previous chemical studies in this genus have informed the presence of coumarins, aromatic acids, monoterpenes and mulinane-type diterpenoids so far [2,3,4,5,6,7]. In the case of *M. crassifolium,* some diterpenoids have been isolated, such as mulinic acid, isomulinic acid, 17-acetoxymulinic acid, mulinolic acid, mulinenic acid, mulin-11,13-dien-20-oic acid, and 17-acetoxymulin-11,13-dien-20-oic acid [3,5,6,7,8,9].

Liquid chromatography coupled to mass spectrometry is considered a powerful method of analysis in environmental, food, and antidoping tests, very important in forensic laboratories as well as in applied research. Analytical challenges and developments in performance have been found not only in ion sources, especially those using atmospheric-to-vacuum interfaces such as atmospheric pressure chemical ionization (APCI), or the most common electrospray (ESI) but also on the increasing use of high-resolution mass spectrometry (HR-MS) instead of low-resolution mass spectra (ion trap MS or triple quadrupole MS). The first HR instruments started with time-of-flight (TOF) instruments and has continued with the most expensive ion cyclotron Fourier transform (ICFT) or Orbitrap mass spectrometers hybridized with quadrupoles. Indeed, Q-TOF and Q-Orbitrap mass analyzers are the most commonly used with HPLC or UHPLC systems. In general, TOF instruments show a mass resolving power of 10,000–60,000 FWHM (full width at half maximum) with a mass accuracy of 1–5 ppm, while the Q-Orbitrap instrument can reach up to 450,000 FWHM at *m*/*z* 200 with <3 ppm mass accuracy. The increased selectivity and accuracy provided by HR-MS is particularly important when studying non-targeted complex organic biological mixtures, such as plants and food extracts. Accurate detection and quantitation rely on the differences between the compounds of interest from background ions and other interferences. Furthermore, the simplification of workflows and fast and flexible scanning functions make LC-HR-MS, particularly Orbitrap MS, suitable for a myriad of applications such as food, drugs, pharmaceuticals, antidoping, environment, and forensic analysis [10]. Orbitrap is particularly useful for the fast analysis of phenolics, fatty acids and diterpenes [11,12].

As part of our studies on mulinane-type diterpenoids from Apiaceae, we describe in this paper the isolation and structural elucidation of two new diterpenoids from *M. crassifolium*. In addition, we discuss here the gastroprotective action of the infusion of this plant (edible form), including a metabolomic profiling of it based on UHPLC-ESI-MS/HRMS for the first time.

## 2. Results and Discussion

### 2.1. Isolation of Diterpenoids

**Figure 1:**
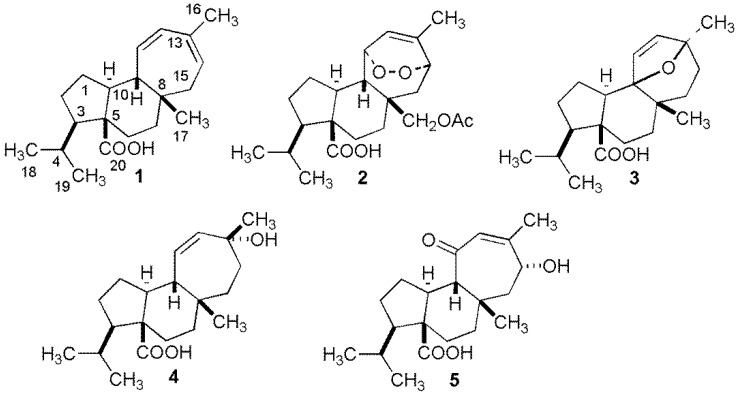
Mulinane diterpenoids isolated from *Mulinum crassifolium*.

The air-dried plant of *M. crassifolium* was extracted with EtOAc at room temperature for nine days. The crude extract obtained after evaporation of the organic solvent was fractionated in four fractions by flash column chromatography (SiO_2_). Each fraction was subjected to repeated permeation with Sephadex LH-20 and chromatography over silica gel to yield two new mulinane-type diterpenoids (**3** and **5**) along with three known diterpenoids (Figure 1).

Compound **3** (Figure 1, 9,13-epoxymulin-11-en-20-oic acid) showed a molecular formula of C_22_H_30_O_3_ (obsd for 317.2127; calcd for 317.2122 [M − H]^−^). This compound showed typical signals of a diterpenoid with a mulinane-type skeleton [6,11]. ^1^H-NMR spectrum showed signals at δ_H_ 0.88 d (6.7; H_3_-18), 1.06 s (H_3_-17), 1.09 d (6.7; H_3_-19), 1.36 s (H_3_-16), 5.65 d (12.6; H-11) and 5.73 d (12.6; H-12). The ^13^C-NMR signals suggested the presence of a carboxyl group at δ_C_ 178.7, two oxygenated carbon at δ_C_ 70.1 and 91.8 (ether bridge), and an alkene at δ_C_ 134.9 (C-11) and 135.1 (C-12). In the HMBC spectrum, the methyl groups of the isopropyl (H_3_-18 and H_3_-19) showed cross peaks with C-3 and C-4, which placed the isopropyl group at position C-3. The methine H-10 showed correlations to C-5 and C-20, which placed the carboxyl group at position C-20. The methyl group H_3_-16 displayed connectivities with a quaternary carbon (C-13) and with carbons C-12 and C-14. In turn, H_3_-17 showed correlations to C-7, C-8, C-9, and C-15. Therefore, the ether bridge was placed at position C-9 and C-13 supported on the cross peak between H-12 and C-9. Analysis of the NOESY spectrum of the **3** did not show dipolar interaction between the signal at δ_H_ 1.36 for H_3_-16 and the H_3_-17 methyl at δ_H_ 1.06 indicating that they were not on the same face of the molecule. No correlation was observed between H-10 and H_3_-17. These data indicated that H-10 and H_3_-17 have α- and β-configuration, respectively. Based on these data, compound **3** was identified as 9,13-epoxymulin-11-en-20-oic acid (Figures S1 and S2; Supplementary Materials).

Compound **5** (Figure 1, 14α-hydroxymulin-12-en-11-one-20-oic acid) was isolated as a yellow gum. HRESIMS and NMR data were consistent with formula molecular of C_20_H_30_O_4_ (*m*/*z* obsd 333.2076; calcd for 333.2071 [M − H]^−^). The ^1^H-NMR spectrum indicated the presence of the typical mulinane isopropyl group at δ=_H_ 0.82 d and 0.97 d (3H each, *J* = 4.9), two methyl groups at δ_H_ 1.02 s and 1.96 s, and one trisubstituted olefine at δ_H_ 5.74 s, which showed to be another mulinane-type diterpenoid. The ^1^H-NMR data were similar to those of 11α-hydroxymulin-12-ene-14-one-20-oic and 14α,17-diacetoxymulin-12-ene-11-one-20-oic acid [9,11]. The ^13^C-NMR data of **5** showed signals at δ_C_ 178.3 s (C-20) and 205.8 s (C-11) corresponding to carboxylic acid and ketone groups, respectively. Two carbons at δ_C_ 125.3 d and 155.1 s confirmed the presence of a trisubstituted double bond in the seven-membered ring, while the presence of a signal at δ_C_ 69.3 was associated to a secondary alcohol. In the HMBC spectrum, the methyl signal at δ_H_ 1.96 s (H_3_-16) showed cross peaks with the carbon signals at δ_C_ 69.3 (C-14), 125.3 (C-12) and 155.1 (C-13), and the proton signal at δ_H_ 5.74 (C-12) had correlations with the carbon signal at δ_C_ 205.8 (C-11), 155.1 (C-13), and 22.2 (C-16) indicating the presence of an α,β-unsaturated ketone. The hydroxyl group position was confirmed by the HMBC correlation of the signal at δ_H_ 1.02 (H_3_-17) with the signal at 69.3 (H-14) confirming the position of the hydroxyl group at C-14. In the NOESY spectrum of **5**, correlation between δ_H_ 4.45 (H-14) and 1.02 (H_3_-17), 1.96 (H_3_-16) and 2.44 (H-9) evidenced that the OH group at C-14 had α-configuration. The experiments sel-pfg-1D NOESY supported these findings. In these experiments, irradiation of H-14β resulted in NOE enhancements of the H_3_-17β and H-15β signals at δ_H_ 1.02 and 2.62, respectively. In turn, irradiation of the H_3_-17β signal resulted in enhancement of the H-14β, H-15β and H-9β signals. Based on these data, compound **5** was identified as 14α-hydroxymulin-12-en-11-one-20-oic acid and had the same relative stereochemistry of previously reported mulinane skeletons (Figures S3 and S4; Supplementary Materials).

### 2.2. Metabolomic Profiling of the Infusion by Using UHPLC-ESI-MS/HRMS

**Figure 2:**
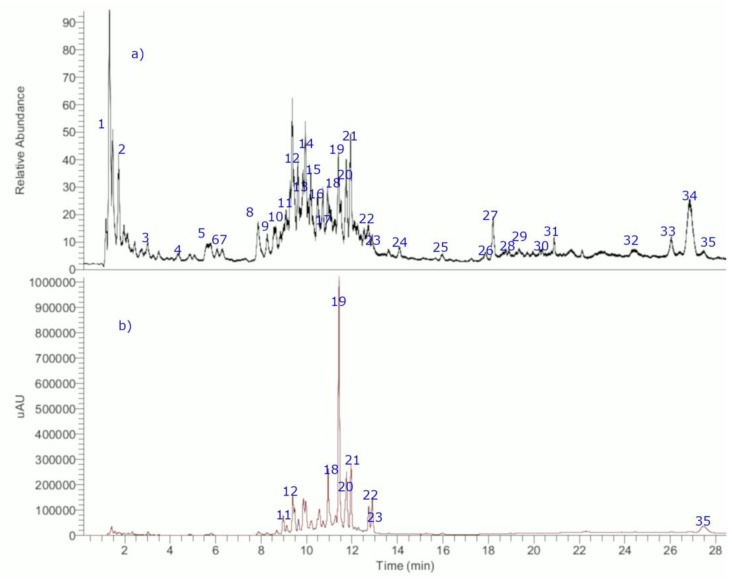
UHPLC chromatrograms of *Mulinum crassifolium*: (**a**) total ion current spectra; and (**b**) UV vis spectra at 280 nm.

The aqueous infusion of chopped *M. crassifolium* Phil. (3.0 g, tea bag) was performed by maceration in 200 mL of distilled water at 85 °C. The edible aqueous infusion was lyophilized to obtain 0.3 g of extract. Afterwards, 10 mg of the extract was re-dissolved in fresh water, filtered and injected in the UHPLC-MS instrument (10 μL) to obtain the chromatograms (Figure 2). This edible extract was chosen for metabolomic profiling due to the high biological activity it showed in the HCl/EtOH-induced gastric lesions model in mice.

#### 2.2.1. Simple Organic Acids

Peak 2 was identified as malic acid (C_4_H_5_O_5_^−^) and peak 3 as citric acid (C_6_H_7_O_7_^−^) [12]. Malic acid is founded naturally in fruits such as apples and pears as well as in vegetables. Citric acid occurs in citrus fruits and is used as an acidifier, flavoring and chelating agent. Both compounds are sold as nutritional supplements and their production is industrial [13].

#### 2.2.2. Phenolic Acids

Peak 1 with a molecular anion at *m*/*z*: 191.05574 was identified as quinic acid (C_7_H_11_O_6_^−^) [14], while several compounds were isomers of caffeoyl quinic acid (C_16_H_17_O_9_^−^). Quinic acid is a cyclic polyol common in plants such as coffee, Tara, Eucalyptus, Urtica, cinchona and so on. Quinic acid is commonly used as astringent. Peak 8 was identified as chlorogenic acid (5-caffeoylquinic acid) [14,15]. Peak 13 as 4-caffeyolquinic acid, peak 15 as 3-caffeyolquinic acid, while peaks 11, 22, 23 and 24 were dicaffeoyl-quinic acid isomers (4,5; 1,3; 1,5 and 1,4 dicaffeoyl-quinic acids, respectively) [16]. Chlorogenic acid is the main phenolic in coffee bean, artichoke, carrot, kiwi fruit, pears, eggplant, peaches, prunes, potatoes, tea, tomatoes, grapes and hibiscus leaves. Chemically, it is an ester formed between caffeic acid moiety and 5-hydroxyquinic acid. Furthermore, the other isomers of chlorogenic acid were detected, showing the caffeoyl-ester attached at different positions on the quinic acid moiety (peak 13 and 15). Chlorogenic acid has showed to be antioxidant, antidiabetic, anti-obesity, antibacterial, hepatoprotective, cardioprotective, anti-inflammatory, antipyretic, neuroprotective, antiviral, anti-microbial, and anti-hypertensive agent, besides central nervous system stimulator [17]. Peaks 26 and 27 were identified as caffeoyl-feruloyl-quinic acid isomers (C_26_H_25_O_12_^−^), peak 18 as *p*-coumaroyl-quinic acid (C_16_H_17_O_8_^−^) and peak 19 as feruloyl-quinic acid (C_17_H_19_O_9_^−^). Peak 10 with an ion at *m*/*z*: 341.08813 was identified as caffeoyl glucoside (C_15_H_17_O_9_^−^) [18]. Some compounds were benzoic acid derivatives [14,19], thus peak 4 with a pseudomolecular ion at *m*/*z*: 329.08801 was identified as 4-*O*-methoxybenzoic acid 3-*O*-glucoside (C_14_H_17_O_9_^−^), peak 5 with a pseudomolecular ion at *m*/*z*: 153.01888 was identified as 3,4-dihydroxybenzoic acid (C_7_H_5_O_4_^−^), peak 6 with a parent ion at *m*/*z*: 315.07251 as 3-*O*-glucosyl-4-hydroxybenzoic acid (C_14_H_17_O_9_^−^) and peak 7 with a molecular anion at *m*/*z*: 299.07748 as 3-*O*-glucosyl-benzoic acid (C_13_H_15_O_8_^−^). Similarly, peak 9 was identified as 3-*O*-di-glucosyl-4-methoxybenzoic acid (C_20_H_27_O_14_^−^) and peak 12 was identified as the derivative 4-*O*-(3-*O*-glucosyl-4-hydroxybenzoyl)-quinic acid (C_20_H_25_O_14_^−^). Peak 14 with an ion at *m*/*z*: 475.14571 was identified as 4-methoxybenzoic acid 3-*O*-rutinoside (C_20_H_27_O_13_^−^) and peak 16 with an ion at *m*/*z*: 359.09872 was identified as syringic acid hexose (C_15_H_19_O_10_^−^) [20]. Finally, peaks 17 and 21 were identified as caffeic and coumaric acids, respectively. The common source of caffeic acid is coffee, wine, oregano, sage apples, olive oil, pears and vegetables. Caffeic acid has many health benefits, including antioxidant, anti-inflammatory, anticancer, and antiviral properties [21]. All acyl-quinic acids identified in our study have been associated with health benefits, including a reduced incidence of several chronic and degenerative diseases, such as cancer, cardiovascular disorders, diabetes, and Parkinson’s disease. Therefore, these protective effects of coffee beans have been ascribed, at least in part, to the acyl-quinic acids present in it [22].

#### 2.2.3. Flavonoids

Peak 20 with a pseudomolecular ion at *m*/*z*: 431.09885 was identified as apigenin 7-*O*-glucoside (C_21_H_19_O_10_^−^) [23]. Similarly, peak 28 was identified as isorhamnetin (C_16_H_11_O_7_^−^) and peak 29 as quercetin (C_18_H_15_O_7_^−^) [24]. Peak 35 was identified as 7,3′,4′-trimethoxyquercetin (C_18_H_15_O_7_^−^). Apigenin-7-*O*-glucoside and apigenin have similar anti-inflammatory capacity and it possess anxiolytic potential [25]. Apigenin has shown attention due to its significant anticancer, antiviral, antibacterial, antioxidant, pro-apoptotic and anti-inflammatory effects [26]. Isorhamnetin, a flavonol aglycone, has demonstrated a variety of biological activities, including antioxidant, anti-inflammatory, antitumor, antiviral, anti-endoplasmic reticulum stress, neuroprotection, and attenuates liver fibrosis by inhibiting transforming growth factor (TGF)β/SMAD signalling [27,28]. Finally, quercetin has showed to reduce gastric ulcer formation [29]. Therefore, the presence of this flavonoid could explain in part their gastroprotective activities.

#### 2.2.4. Fatty Acids

Peak 31 showing an ion at *m*/*z* 271.22803 was tentatively identified as hydroxypalmitic acid (C_16_H_31_O_7_^−^).

#### 2.2.5. Mulinane Diterpenoids

Peak 30 with an ion at *m*/*z*: 335.22302 was identified as 13,14-dihydroxymulin-11-en-20-oic acid (C_20_H_31_O_4_^−^), while peak 32 was identified as 14α-hydroxymulin-12-en-11-one-20-oic acid (by NMR and spiking experiment with authentic compound **5**). In addition, peak 33 was identified as 14α-acetoxymulin-12-en-11-one-20-oic acid (C_22_H_33_O_5_^−^) [3,6,9,30] and peak 34 as 9,15-epoxymulin-11-en-20-oic acid (by spiking experiments with the authentic compound, which was isolated as compound **3** and elucidated by NMR).

### 2.3. Gastroprotective Activity

The two new mulinane diterpenoids were not evaluated as gastroprotective agents since the amount isolated was not enough. However, the organic extract (EtOAc-E) and edible infusion (INF-E) showed gastroprotective effect at 100 mg/kg in HCl/EtOH induced gastric ulcer model in mice, reducing lesions by 33% and 74%, respectively (Table 1). This dose was taken in account based on traditional medicine and to validate their ethnopharmacological effectiveness of these crude extracts. Thus, the gastroprotective effect of the edible infusion was related to the presence of secondary metabolites, mainly phenolics and some diterpenes, which were detected using UHPLC-ESI-MS/HRMS.

During the last century, many reports have been published in relation to new gastroprotective agents from natural sources. Some examples such as carbenoxolone from *Glycyrrhiza glabra*, solon from sophoradin and gefarnate from cabbage have been marketed [31]. Some mulinane-type diterpenoids from *Azorella* species, desertic shrubs that are closely related to *M. crassifolium*, and are generally called “Yaretas” by local people, have shown gastroprotective activity on the HCl/EtOH-induced gastric lesion model in mice. Among the compounds studied, azorellanol, 13β-hydroxyazorellane and mulin-11,13-dien-18-acetoxy-16,20-dioic acid showed gastroprotective effect, being as active as lansoprazole and reducing the gastric lesions by at least 69%. The same study demonstrated that the mulinane diterpenoids have increased the activity due to the presence of an additional carboxylic acid [32]. In another study, the hydro-carbonated compounds mulin-11,13-diene, mulin-11,13-dien-20-ol, and mulin-11,13-dien-20-oic acid were less active than lansoprazole reducing the gastric lesions by 18%, 26% and 39%, respectively [32,33]. Regarding the mode of gastroprotective action for mulin-11,13-dien-20-oic acid evaluated at 55 mg/kg, its effect was reduced by pre-treatment with indomethacin and *N*-ethylmaleimide, suggesting that prostaglandins and sulfhydryl compounds are positively involved in the gastroprotective activity of this mulinane diterpenoid [11]. Taking into consideration that many crude drugs have shown gastroprotective activity [29,34,35,36], we decided to evaluate the edible extracts obtained from *M. crassifolium* since it is used in folk medicine to treat stomach sickness [1]. Metabolomic profiling of the edible infusion (74% gastroprotection) shows the presence of polyphenols and terpenoids (Table 2) and it is well known that phenolics have a beneficial role in disease prevention as well as in gastroprotection [29,35]. Moreover, it has been suggested that phenolic compounds stimulate PGE_2_ formation, decrease of histamine secretion, and inhibit *H. pylori*, which explain the gastroprotective effect [29,37,38]. The presence of those compounds in the infusion of *M. crassifolium* associated to the gastroprotective effect could be due to the synergistic effect produced by the compounds detected. In fact, quercetin (peak 27), caffeic acid (peak 17), apigenine glucoside (peak 20), *p*-coumaric acid (peak 21), chlorogenic acid (peak 8), and caffeoylquinic acids (peaks 11, 13, 15 and 23–25) have been associated with ulcer preventing capacity [39,40,41,42]. We thus demonstrated that the edible infusion at 100 mg/kg had gastroprotective effect, which could be linked to the presence of several phenolic compounds that could increase the activity of the diterpenoids, but further studies are required to isolate and evaluate the individual gastroprotection of these compounds.

## 3. Materials and Methods

### 3.1. Chemicals

TLC (Kieselgel 60 GF_254_, Merck) were developed in *n*-hexane/EtOAc mixtures and spots were revealed by spraying plates with H_2_SO_4_-MeOH (5:95, *v*/*v*) and heating at 120 °C. Silica gel (Kieselgel 60, Merck, Santiago, Chile 0.063–0.200 mm) and Sephadex (LH-20, Sigma Aldrich, Santiago, Chile) were used in column chromatography (CC). Technical solvents used in chromatography processes were previously distilled and dried according to standard procedures. Caffeic acid, *p*-coumaric acid, 1,5-Dicaffeoylquinic acid, isorhamnetin, and quercetin (purity 95%, by HPLC) were acquired in Extrasynthese (Genay, France) or Sigma Aldrich (Santiago, Chile).

### 3.2. Plant Material

*Mulinum crassifolium* were collected in 2016 at “El Tatio” (Antofagasta, II Región, Chile) and identified by Prof. J. Bórquez. A voucher specimen (Nº 160316) is kept at the Natural Product Lab of the University of Antofagasta.

### 3.3. Extraction and Isolation

Dried and pulverized aerial parts of *M. crassifolium* (0.8 kg) were macerated with ethylacetate (3 times, 2.0 L, 3 day/extraction). After filtration, the organic solvent was concentrated under reduced pressure yielding an extract (EtOAc-EXT 20 g). This extract (20 g) was submitted to flash chromatography on silica gel (63–200 μm, 200 g, column length 25 cm, i.d. 10 cm) and eluted with *n*-hexane–EtOAc mixtures (2 L each) of increasing polarity (8:2, 6:4, 4:6, and 0:1; *v*/*v*) to produce four fractions.

Fraction 1 (10 g, *n*-hexane–EtOAc, 8:2) was submitted to CC and eluted with EtOAc–*n*-hexane (0:1, 0.5:9.5, 1:9, 1.5:8.5, and 2:8 *v*/*v*) affording 100 subfractions (25 mL each). By TLC comparison, they were joined into three main fractions (1A–1C). CC (silica gel 63–200 μm, 50 g) on Fraction 1A (2 g) eluted with *n*-hexane–EtOAc mixtures (0–5% EtOAc) led to the isolation of compounds **1** (mulin-11,13-dien-20-oic acid, 400 mg) [7]. Fractions 1B and 1C (3.0 g and 2.0 g) were submitted to Sephadex LH-20 (column length 40 cm, i.d. 6.5 cm) using ternary mixtures (3 L, *n*-hexane–DCM–MeOH, 3:2:1) to afford mulin-11,13-dien-20-oic acid **1** (300 mg), and lipids according to ^1^H-NMR.

Fraction 2 (4.0 g, *n*-hexane–EtOAc 6:4) was purified on Sephadex LH-20 using MeOH as mobile phase allowing the separation of fatty acid, chlorophylls and pigments from the diterpenoids. CC on silica gel using *n*-hexane–EtOAc mixtures (9:1, 8:2, 1:1, and 0:1 *v*/*v*) gave four fractions (2A–2D). Further CC on Fraction 2A (1.0 g) using silica gel afforded compound **2** (17-acetoxymulinic acid, 100 mg) [3] and the new compound **3** (10 mg). Fraction 2B (0.3 g) was purified using CC on silica gel, affording compound **4** (mulinolic acid, 90 mg) (Loyola et al., 1996) and the new compound **5** (5.0 mg). ^1^H-NMR analysis of other fractions suggested that they were not diterpenoids.

Fraction 3 (2.0 g, *n*-hexane–EtOAc, 4:6) and Fraction 4 (1.0 g, *n*-hexane–EtOAc, 0:1) were submitted to CC using Sephadex LH-20 (MeOH) but did not contain mulinane-type diterpenoids, as shown by TLC and ^1^H-NMR analysis.

9, 13-epoxymulin-11-en-20-oic acid (**3**): colorless gum; FT-IR ν_max_: 3300–2600, 1700, 1639, and 1205 cm^−1^; HRESIMS (negative mode) *m*/*z* 317.2099 [M − H]^−^ (calcd. for C_20_H_29_O_3_: 317.2122).

14α-hydroxymulin-12-en-11-one-20-oic acid (**5**): yellow gum; FT-IR ν_max_: 3300, 1710, 1660, 1490, 1205, 1025 cm^−1^; HRESIMS (negative mode) *m*/*z* 333.2076 [M − H]^−^ (calcd. for C_20_H_29_O_4_: 333.2071).

### 3.4. UHPLC-ESI-MS/HRMS Studies

For this study, 3.0 g of dried and chopped aerial parts were macerated with ethyl acetate (3 times, 50 mL at 25 °C), and an infusion prepared using 3.0 g of dried chopped aerial parts adding deionized water (200 mL) at 85 C, for 1 h. The solvents were concentrated in vacuo at 45 °C and the infusion lyophilized (Labconco, MA, USA) to obtain 125, and 300 mg of EtOAc and edible aqueous extracts, respectively.

A UHPLC Thermo Scientific Ultimate 3000, with all accessories including a quaternary pump, TCC-3000RS vial compartments, WPS-3000RS autosampler and PDA detector with Chromeleon 7.2 Software (Thermo Fisher Scientific, Germany) was connected to a Thermo high- resolution Q Exactive focus spectrometer (Thermo, Bremen, Germany) by means of an Electrospray Ionization Source II (HESI II). The nitrogen was produced by a Genius NM32LA nitrogen generator (purity > 99.999%, Peak Scientific, Billerica, MA, USA) and used as both damping and collision gas. Mass calibration for Orbitrap was performed every day, in both negative and positive modes, to ensure a mass accuracy lower than 5 ppm.

Chromatography was run using a C18 column (25 mm ID, Acclaim, 150 mm × 4.6 mm, Thermo Fisher Scientific, Bremen, Germany) at 25 °C. The detection used 354, 254, 280, and 330 nm, and PDA recorded from 200 to 800 nm. Mobile phases were formic aqueous formic acid (0.1% A) and acetonitrile formic acid 0.1% (B). The gradient program started at 5% B, maintaining for 5 min, then up to 15% B for 10 min, then to 30% B until 15 min, then 70% B until 25 min, then coming back to 5 min at 35 min., and waiting 12 min using 5% B for column equilibration before each injection, at a flow rate of 1.00 mL min^−1^, using an injection volume of 10 µL. Standards and the extracts were kept in vials at 10 °C during storage.

The HESI parameters were: 75 units of sheath gas flow rate; 20 units of auxiliary gas unit flow rate; 400 °C of capillary temperature; 500 °C auxiliary gas heater temperature; 2500 V of spray voltage; S lens; and RF level 30. Full scan data were acquired at 70,000 FWHM (full width half maximum) at *m*/*z* 200. A scan range of *m*/*z* 100–1000 was chosen; the automatic gain control (AGC) was set at 3 × 10^6^ and the injection time was set to 200 ms. The scan-rate was set at 2 scans s^−1^. External calibration was performed using a calibration solution in positive and negative modes, with the Orbitrap spectrometer operating both in positive and negative modes at 17,500 FWHM (*m*/*z* 200). The AGC target was set to 2 × 10^5^, with injection time of 20 ms. The ultrahigh vacuum, fore vacuum and high vacuum and were maintained at 1010, 2, and 105 mbar, respectively. Collision energy in the HCD cell was operated at 30 kv. Detection operated on calculated exact mass and on retention time of compounds, as shown in Table 2. The tolerance window for mass detection was set to 5 ppm for the two modes.

### 3.5. Animals

Swiss albino mice were acquired at the Instituto de Salud Pública de Chile, Santiago, Chile. Mice weighing 30 ± 3 g were fasted for 24 h before the tests. The animals were fed on Champion certified diet with access to free water under 12 h dark–light period at standard conditions, 50% humidity and 22 °C of temperature. The protocols were approved by the Universidad de Chile (Animal Care Committee) that follows the recommendations of the Canadian Council on Animal Care and with the ethical guidelines for investigations in conscious animal.

### 3.6. HCl/EtOH-Induced Lesions in Mice

The gastroprotective activity of the extracts was assessed in the HCl/EtOH-induced lesion model, as already described [43]. Mice were randomly distributed into groups of seven animals each and fasted for 24 h with free access to water prior to the experiment. Fifty minutes after oral administration of the extracts (100 mg/kg), lansoprazole (30 mg/kg) or water (control), all groups were treated orally with 0.2 mL of a solution containing 0.3 M HCl/60% ethanol (HCl/EtOH) for the induction of gastric lesion. Approximately 1 h after the administration of HCl/EtOH, Animals were sacrificed and the stomachs were inflated and excised by injection of 1 mL of saline solution. A concentration of 5% formalin was used to fix the ulcerated stomachs for 30 min, which were opened along the greater curvature. Visible gastric damage was then observed in the gastric mucosa as elongated black-red lines, parallel to the long axis of the stomach similar to the HCl/EtOH-induced lesions in rats. The length in mm of each lesion was measured, and the sum of the length of all lesions was expressed as lesion index.

### 3.7. Statistical Analysis

Results were expressed as the mean ± S.D. In all experiments, statistical differences between treatments and their respective control were determined by one-way analysis of variance (ANOVA) followed by Dunnett’s test. The level of significance was set at *p* < 0.01. All statistical analyses were performed using the software GraphPad Prism 4 for Windows.

## 4. Conclusions

In the present study, we isolated two new mulinane-type diterpenoids from the ethyl-acetate extract from *M. crassifolium* using a combination of chromatographic techniques and identification by NMR. In addition, we prepared an infusion (edible aqueous form) and both organic and water extracts were evaluated as gastroprotective agents using the HCl/EtOH-induced gastric lesion model in mice. Bioactivity assay results show that the infusion protected the gastric lesions by 74%, while the organic extract by 33%. Finally, to correlate the gastroprotective activity with the presence of bioactive compounds, some UHPLC-ESI-MS/HRMS analysis was performed. Thirty-five compounds were tentatively identified, being mostly polyphenolics the main compounds. Among the compounds identified, quercetin, caffeic acid, apigenine glucoside, *p*-coumaric acid, chlorogenic acid, and caffeoylquinic acids have been previously associated with gastroprotective and antiulcer properties. Gastric ulcers are becoming a serious public health problem due to stress and other factors and new therapeutic agents for the prevention of them are necessary. Our findings based on traditional medicine provide the basis for the utilization of *M. crassifolium* as a source of potential compounds for the prevention of gastric ulcers. Moreover, these extracts can be utilized as a natural alternative to synthetic gastroprotective agents for the use in the current therapy.

## Figures and Tables

**Table 1:** Gastroprotective effect of the organic (EtOAc-E) and edible extract (INF-E) on HCl/EtOH-induced gastric lesions in mice.

Compound	*n*	Lesion Index (mm)	% Lesion Reduction	Dose (mg/Kg)
EtOAc-E	7	40.6 ± 1.5 **	33 *	100
INF-E	7	15.5 ± 1.1	74 *	100
Lansoprazole	7	12.9 ± 2.9	78 *	30
Control	7	60.6 ± 1.9	-	-

The results are expressed as mean ± sem * *p* < 0.01, significantly different compared with the control; ** *p* < 0.01, significantly different compared with lansoprazole (ANOVA followed by Dunnett’s test). *n* = number of mice.

**Table 2:** UHPLC/ESI/MS spectral data of edible infusion of *Mulinum crassifolium*.

Peak #	RT (min)	UV Max	Tentative Identification	[M − H]^−^	Theoretical Mass (*m*/*z*)	Measured Mass (*m*/*z*)	Accuracy (δppm)	MS Ions
1	1.35	-	Quinic acid	C_7_H_11_O_6_^−^	191.05611	191.05574	−1.93	109.02866 (C_6_H_5_O_2_^−^); 173.04552 (C_7_H_9_O_5_^−^)
2	1.45	-	Malic acid	C_4_H_5_O_5_^−^	133.01425	133.01364	−4–59	115.00294 (C_4_H_3_O_4_^−^, M^−^-H_2_O)
3	1.87	210	Citric acid	C_6_H_7_O_7_^−^	191.01973	191.01938	−1.83	111.00797 (C_5_H_3_O_3_^−^)
4	2.42	245	3-*O*-glucoside-4-methoxybenzoic acid	C_14_H_17_O_9_^−^	329.08781	329.08801	0.60	167.0345 (C_8_H_7_O_4_^−^)
5	3.47	245	3,4-dihydroxybenzoic acid	C_7_H_5_O_4_^−^	153.01933	153.01888	−2.94	109.02893 (C_6_H_5_O_2_-)
6	4.35	245	3-*O*-glucosyl-4-hydroxybenzoic acid	C_14_H_17_O_9_^−^	315.07216	315.07251	1.11	153.01856 (C_7_H_5_O_4_^−^)
7	5.75	245	3 or 4-*O*-glycosylbenzoic acid	C_13_H_15_O_8_^−^	299.07724	299.07748	0.80	137.0236 (C_7_H_5_O_3_^−^)
8	7.85	236–329	Chlorogenic acid, (1- Caffeoylquinic acid)	C_16_H_17_O_9_^−^	353.08781	353.08813	0.90	191.05559 (C_7_H_11_O_6_^−^) Quinic acid
9	8.23	236–329	3-*O*-diglucosyl-4-methoxy-3-hydroxybenzoic acid	C_20_H_27_O_14_^−^	491.14063	491.14096	0.67	167.0341(C_8_H_7_O_4_^−^)
10	8.62	236–325	Caffeoyl glucoside	C_15_H_17_O_9_^−^	341.08781	341.08813	0.93	179.0564 (C_6_H_11_O_6_^−^); 135.0445 (C_8_H_7_O_2_^−^)
11	8.97	236–329	4,5-dicaffeoylquinic acid	C_22_H_27_O_14_^−^	515.14063	515.14087	0.46	191.05559 (C_7_H_11_O_6_^−^) Quinic acid
12	9.09	245	4-*O*-(3-*O*-glucosyl-4-hydroxybenzoyl)-quinic acid	C_20_H_25_O_14_^−^	489.12498	489.12521	0.47	191.05586 (C_7_H_11_O_6_^−^) Quinic acid
13	9.36	236–329	3-Caffeoylquinic acid	C_16_H_17_O_9_^−^	353.08781	353.08820	1.10	191.05579 (C_7_H_11_O_6_^−^) Quinic acid
14	9.44	245	4-Methoxybenzoic acid 3-*O*-rutinoside	C_20_H_27_O_13_^−^	475.14571	475.14571	0.0	151.03946 (C_8_H_7_O_3_^−^)
15	10.62	236–329	5-caffeoylquinic acid×	C_16_H_17_O_9_^−^	353.08781	353.08820	1.10	191.05579 (C_7_H_11_O_6_^−^) Quinic acid
16	10.86	279	Syringic acid hexoside	C_15_H_19_O_10_^−^	359.09837	359.09872	0.97	197.0445 (C_9_H_9_O_5_^−^)
17	10.95	236–325	Caffeic acid×	C_9_H_7_O_4_^−^	179.03498	179.03470	−1.56	135.04457 (C_8_H_7_O_2_^−^)
18	11.18	236–329	*p*-Coumaroylquinic acid	C_16_H_17_O_8_^−^	337.09289	337.09320	0.91	191.05573 (C_7_H_11_O_6_^−^) Quinic acid
19	11.28	236–329	Feruloyl-quinic acid	C_17_H_19_O_9_^−^	367.10346	367.10391	1.22	191.05577 (C_7_H_11_O_6_^−^) Quinic acid
20	11.62	267–335	Apigenin 7-*O*-glucoside	C_21_H_19_O_10_^−^	431.09837	431.09885	1.11	269.0459 (C_15_H_9_O_5_^−^) apigenin
21	11.76	233–325	*p*-Coumaric acid×	C_9_H_7_O_3_^−^	163.04007	163.03973	−2.08	119.04955 (C_8_H_7_O_3_^−^, M^−^-CO_2_)
22	12.56	254–354	Isorhamnetin-3-*O*-rutinoside	C_16_H_11_O_7_^−^	623.16162	623.16162	0.00	315.05118
23	12.90	236–329	1,3-Dicaffeoylquinic acid	C_25_H_23_O_12_^−^	515.11950	515.11981	0.60	135.04446 (C_8_H_7_O_2_^−^, caffeic acid-CO_2_); 173.04506 (C_7_H_9_O_5_^−^, quinic acid-H_2_O)
24	14.76	236–329	1,5-Dicaffeoylquinic acid×	C_25_H_23_O_12_^−^	515.11950	515.11969	0.36	135.04454 (C_8_H_7_O_2_^−^); 191.05576 (C_7_H_11_O_6_^−^)
25	16.92	236–329	1,4-Dicaffeoylquinic acid	C_25_H_23_O_12_^−^	515.11950	515.11975	0.48	135.04454 (C_8_H_7_O_2_^−^); 173.04509 (C_7_H_9_O_5_^−^)
26	17.34	236–329	caffeoylferuloylquinic acid	C_26_H_25_O_12_^−^	529.13515	529.13544	0.54	135.04449 (C_8_H_7_O_2_^−^); 173.04501 (C_7_H_9_O_5_^−^)
27	18.12	236–329	Caffeoyl-feruloyl-quinic acid	C_26_H_25_O_12_^−^	529.13515	529.13538	0.43	135.04449 (C_8_H_7_O_2_^−^); 173.04501 (C_7_H_9_O_5_^−^)
28	18.44	254–354	Isorhamnetin×	C_16_H_11_O_7_^−^	315.05103	315.05136	1.04	300.02731 (C_15_H_8_O_7_^−^, M-CH_3_)
29	19.14	254–354	Quercetin×	C_18_H_15_O_7_^−^	301.03538	301.03571	1.09	151.00342 (C_8_H_3_O_4_^−^)
30	19.96	18.20	13,14-Dihydroxymulin-11-en-20-oic acid	C_20_H_31_O_4_^−^	335.22278	335.22302	0.71	No diagnostic ions known
31	20.89	217	Hydroxy-palmitic acid	C_16_H_31_O_7_^−^	271.22787	271.22803	0.58	No diagnostic ions known
32	24.52	-	14α-Hydroxy-mulin-12-en-11-one-20-oic acid (compound 5)	C_20_H_29_O_4_^−^	333.20718	333.20758	1.20	135.04778
33	26.34	211	14α-acetoxy-mulin-12-en-11-one-20-oic acid	C_22_H_33_O_5_^−^	377.23380	377.23335	−1.19	323.32305
34	27.36	245	9,13-Epoxymulin-11-en-20-oic acid (compound 3)	C_20_H_29_O_4_^−^	317.21223	317.21273	−1.7	No diagnostic ions known
35	27.78	255–355	7,3′,4′-Trimethoxy-quercetin	C_18_H_15_O_7_^−^	343.08233	343.082276	−0.15	179.0432

MS = Daughter ions. × Identity confirmed using co-spiking experiments with authentic compounds.

## Supplementary Materials

The following are available online.
