# Supplementary material for: Mulinum crassifolium Phil; Two New Mulinanes, Gastroprotective Activity and Metabolomic Analysis by UHPLC-Orbitrap Mass Spectrometry
Source: Molecules. 2019 Apr 28;24(9):1673. doi: 10.3390/molecules24091673 (PMC6539732; doi:10.3390/molecules24091673)
Supplement: Supplementary file 1 [file molecules-24-01673-s001.pdf]

Supplementary Material

Mulinum crassifolium Phil; Two new mulinane, gastroprotective activity and metabolomic analysis by UHPLC-Orbitrap mass spectrometry

Carlos Areche[1], Ronald Fernandez-Burgos[1,2], Teresa Cano[2], Mario Simirgiotis[3], Olimpo García-Beltrán[4], Jorge Borquez[5], Beatriz Sepulveda[6*]

[1]Departamento de Química, Facultad de Ciencias, Universidad de Chile, Santiago, Chile

[2]Departamento de Química, Facultad de Ciencias Naturales y Formales, Universidad Nacional de San Agustín, Arequipa, Perú

[3]Instituto de Farmacia, Facultad de Ciencias, Universidad Austral de Chile, Valdivia, Chile

[4]Facultad de Ciencias Naturales y Matemáticas, Universidad de Ibagué, Carrera 22 calle 67, Ibagué 730002, Colombia

[5]Departamento de Química, Facultad de Ciencias Básicas, Universidad de Antofagasta, Av Coloso S-N, Antofagasta, Chile

[6]Departamento de Ciencias Químicas, Universidad Andres Bello, Campus Viña del Mar, Quillota 980, Viña del Mar, Chile

*** Correspondence:** bsepulveda@uc.cl

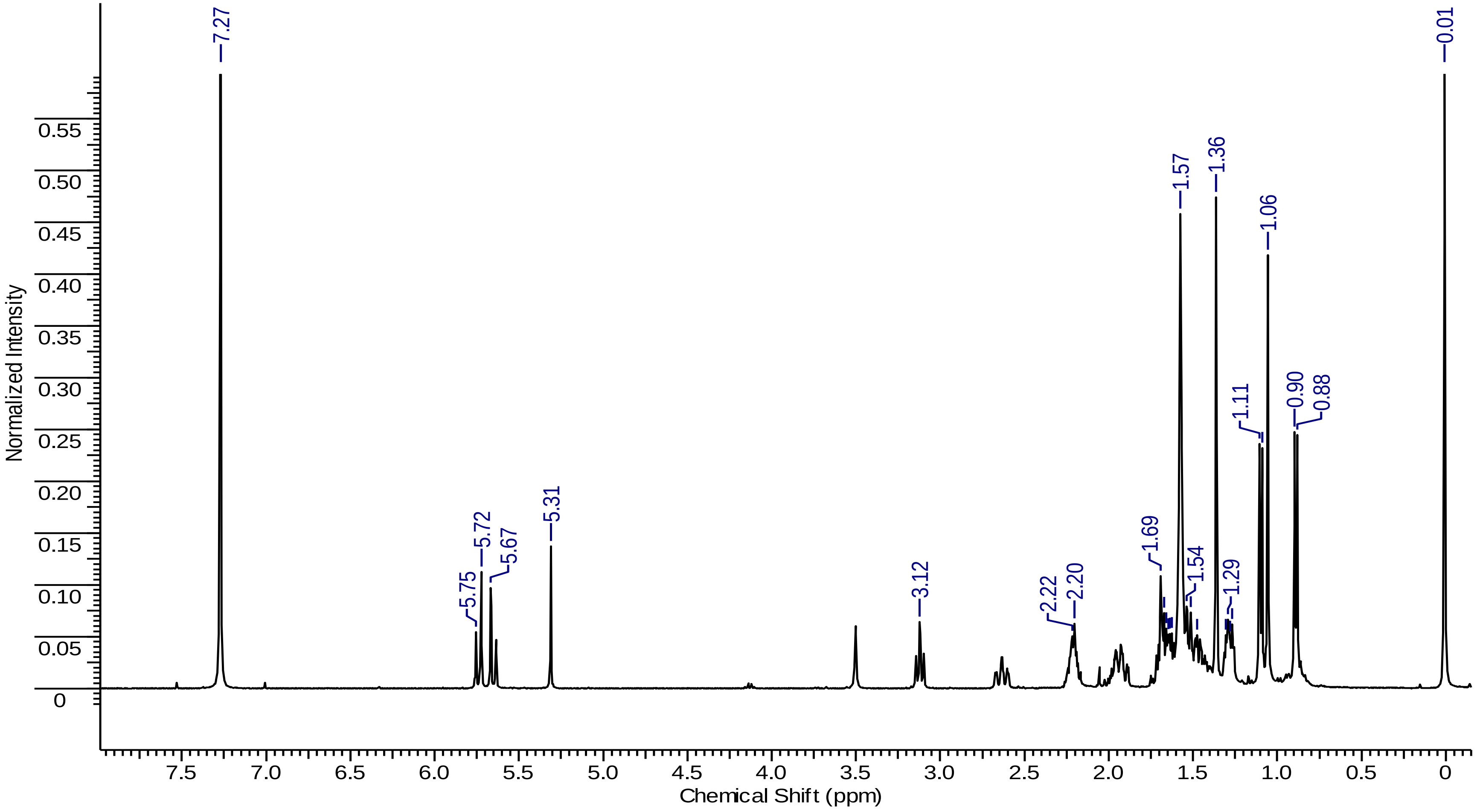

S1. ^{1}H NMR spectrum of the compound **3** (400 MHz, $CDCl_3$)

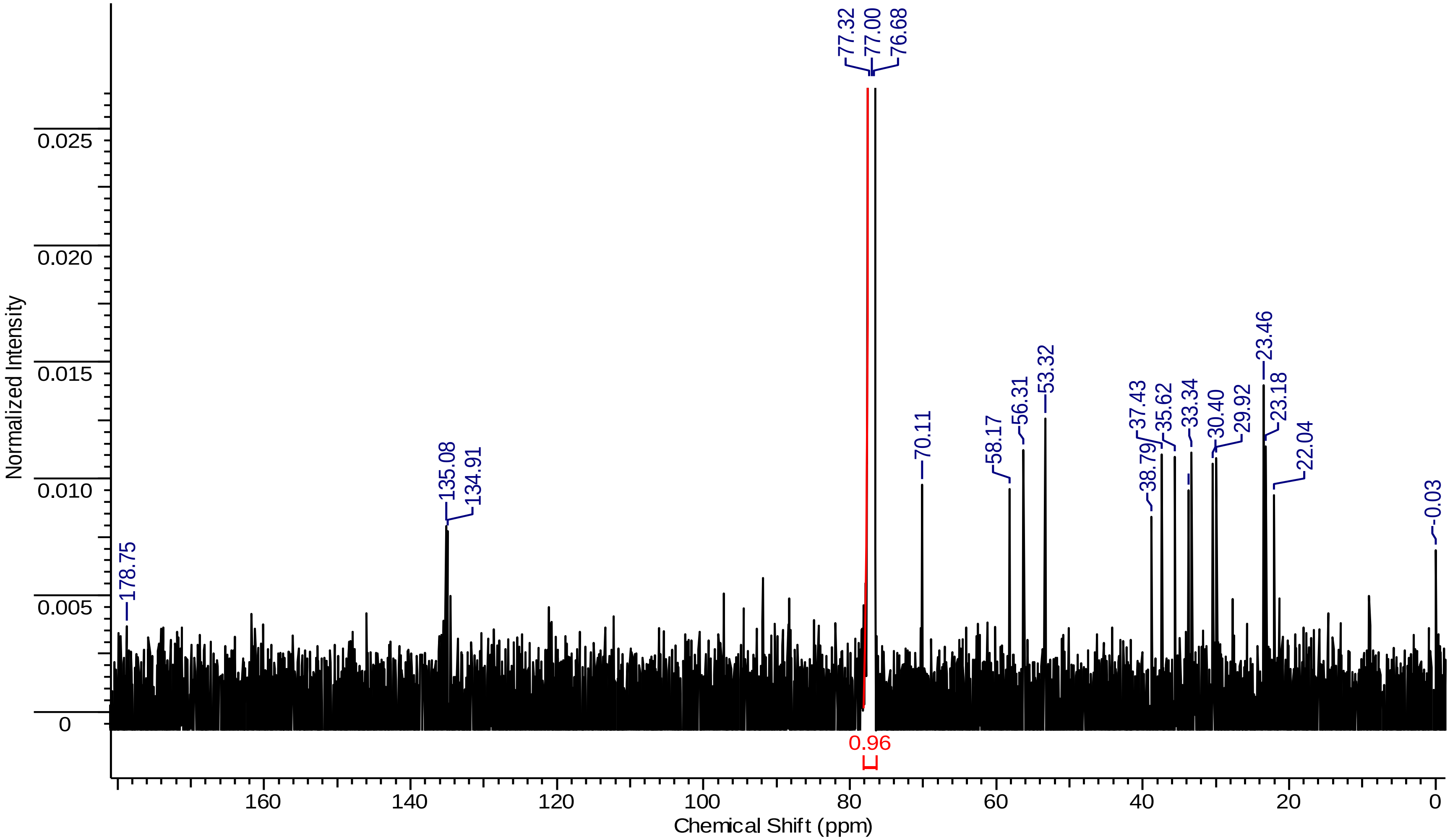

S2. ^{13}C NMR spectrum of the compound **3** (100 MHz, $CDCl_3$)

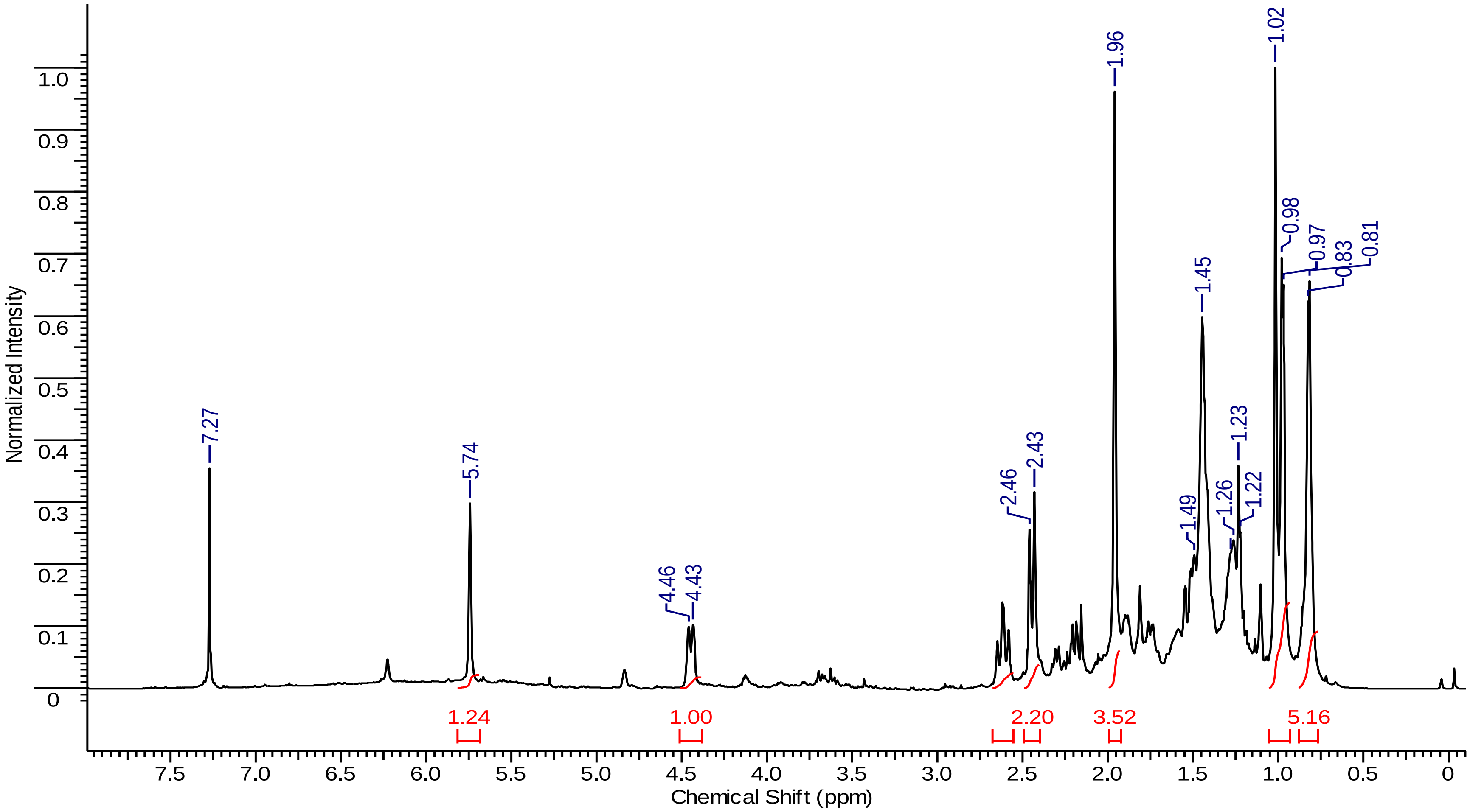

S3. ^{1}H NMR spectrum of the compound **5** (400 MHz, $CDCl_3$)

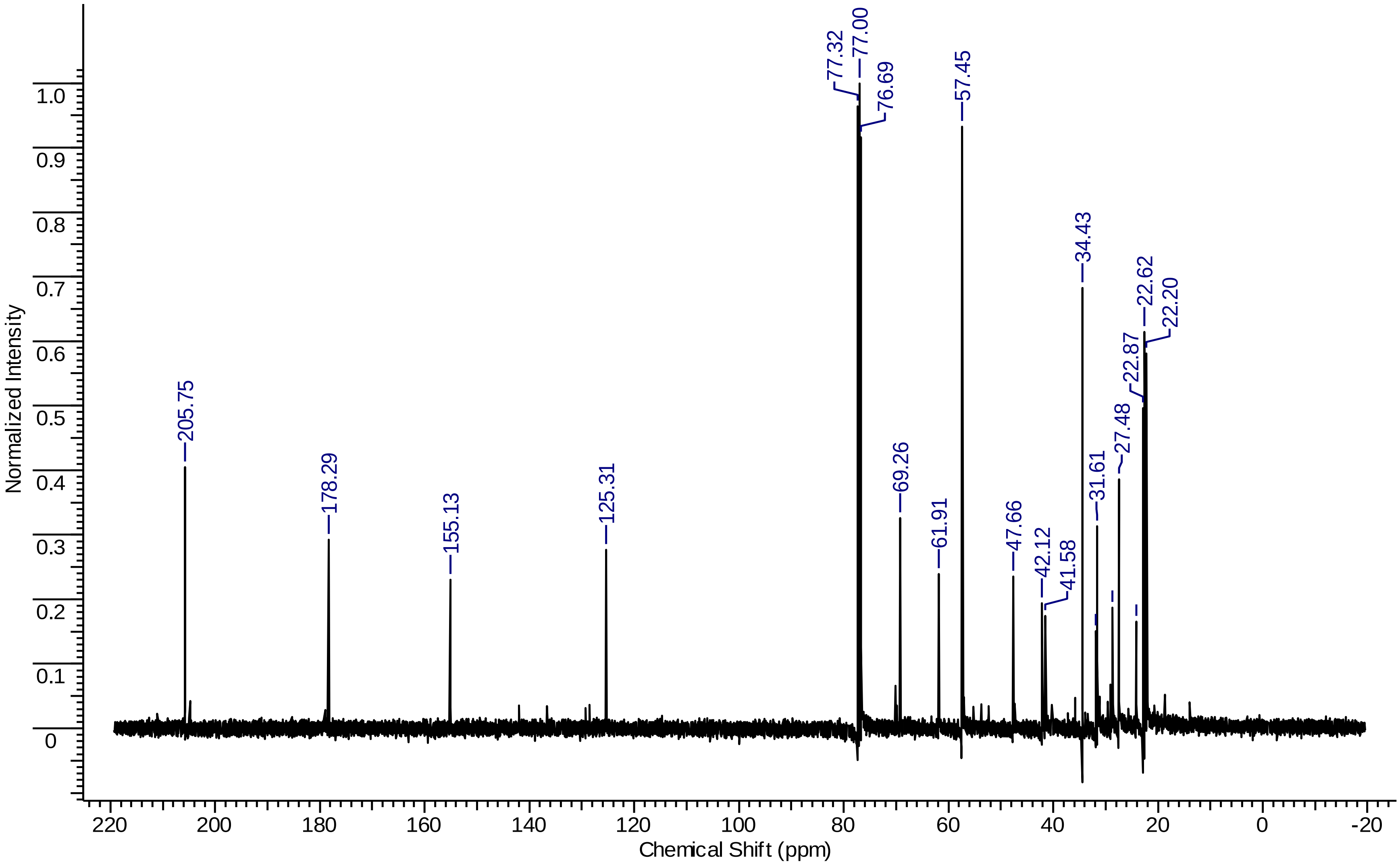

S4. ^{13}C NMR spectrum of the compound **5** (100 MHz, $CDCl_3$)
